# Investigating the Health Potential of *Mentha* Species Against Gastrointestinal Disorders—A Systematic Review of Clinical Evidence

**DOI:** 10.3390/ph18050693

**Published:** 2025-05-08

**Authors:** Mariana Hirata, Lucas Fornari Laurindo, Victória Dogani Rodrigues, Flávia Cristina Castilho Caracio, Vitor Engrácia Valenti, Eliana de Souza Bastos Mazuqueli Pereira, Rodrigo Haber Mellem, Cláudia Rucco Penteado Detregiachi, Manuela dos Santos Bueno, Leila Maria Guissoni Campos, Caio Sérgio Galina Spilla, Sandra Maria Barbalho

**Affiliations:** 1 Department of Biochemistry and Pharmacology, School of Medicine, Universidade de Marília (UNIMAR), Marília 17525-902, SP, Brazil; 2 Department of Biochemistry and Pharmacology, School of Medicine, Faculdade de Medicina de Marília (FAMEMA), Marília 17519-030, SP, Brazil; 3 Autonomic Nervous System Center, Universidade Estadual Paulista (UNESP), Marilia 17525-900, SP, Brazil; 4 Postgraduate Program in Structural and Functional Interactions in Rehabilitation, School of Medicine, Universidade de Marília (UNIMAR), Marília 17525-902, SP, Brazil; 5 Department of Biochemistry and Nutrition, School of Food and Technology of Marília (FATEC), Marília 17500-000, SP, Brazil

**Keywords:** *Mentha*, *Mentha* species, dyspepsia, abdominal pain, gastrointestinal disorders, inflammation, oxidative stress, medicinal plant, phytochemical, bioactive compounds

## Abstract

**Background/Objectives:** Gastrointestinal disorders include a broad spectrum of clinical conditions due to various symptoms. Abdominal pain claims attention as it can be associated with multiple diseases, and some of them can lead to chronic abdominal pain, such as chronic gastritis and irritable bowel syndrome. Moreover, dyspepsia is also a prevalent condition, and its symptoms are postprandial fullness, epigastric pain or burn, and early satiety. Conventional therapeutic approaches for gastrointestinal disorders exist, but the *Mentha* plant has a millenary tradition. *Mentha* aerial parts and leaves hold therapeutic and pharmacological value, and its components are characterized as non-essential oil with superabundant phenolic compounds, and essential oil classified as volatile secondary metabolites like menthol and menthone. Studies have shown that *Mentha* species can exert benefits by modulating the inflammatory process and scavenging free radicals, which can benefit gastrointestinal tract disorders. The aim of this review was to systematically investigate the effects of *Mentha* species on gastrointestinal disorders. **Methods:** Sixteen clinical trials included patients diagnosed with irritable bowel syndrome, functional dyspepsia, and functional abdominal pain, as well as some healthy volunteers. The COCHRANE tool was utilized to assess the bias of the included studies. **Results:** Most studies reported significant outcomes for *Mentha* oil-treated groups, such as better control of abdominal pain and discomfort, even though two trials did not report superior outcomes. **Conclusions:** Due to the increasing interest in natural compounds, further clinical trials are necessary to confirm the status of *Mentha* for improvement in gastrointestinal disorders.

## 1. Introduction

Gastrointestinal diseases have many clinical conditions, and their symptoms are highly prevalent. Most of these people will probably have no organic reason for their symptoms. Consequently, they will be classified as patients with a functional gastrointestinal disorder, which can be dyspepsia, irritable bowel syndrome (IBS), or constipation. Functional gastrointestinal disorders are considered heterogeneous due to the number of symptoms described as well as the underlying pathophysiological mechanisms of the human body, which is highly complex as it involves gut–brain interaction and its bidirectional dysregulation, visceral hypersensitivity, alteration of the mucosal immune function, and abnormal gastrointestinal motility. As far as the biopsychosocial model says, the interaction among biological, psychological, and social factors greatly influences the predisposition to the initiation and course of the disease [1,2,3,4,5,6].

Abdominal pain is a complaint that challenges both primary care workers and gastroenterologists. Due to its extensive differential diagnosis, multiple aetiologies, and the spectrum of symptoms that frequently lead to diagnostic and therapeutic dilemmas, it can be associated with various diseases. Furthermore, an incorrect diagnosis might be made because of the problematic correlation to a particular organ pathology, as the pain occurs at multiple locations with a diversity of intensities and propagation; even though the pain may originate from within the peritoneal cavity, the pelvis, the abdominal wall, the retro peritoneum, or even from outside the abdomen. Although most cases of abdominal pain are benign, consistent numbers of patients are reported to have life-threatening conditions, since the abdominal pain can be chronic, as the pain persists for three months or more. In addition to it, a variety of diseases are responsible for chronic abdominal pain, such as chronic gastritis, Crohn̕s disease (CD), chronic pancreatitis, functional dyspepsia (FD), and IBS [7,8,9,10].

Dyspepsia is a Greek term that means “bad digestion”. It refers to the gastroduodenal location of the gastrointestinal tract and its complex symptoms despite being a prevalent condition. Around 80% of the people with dyspepsia have no organic explanation for their symptoms. They are said to have FD, as the symptoms included are postprandial fullness, epigastric pain or burn, and early satiety. The pathophysiology mechanism of FD is multifactorial and not completely understood. Still, it is already known that some risk factors are considered, such as female sex, acute gastroenteritis, psychological comorbidity, use of non-steroidal anti-inflammatory drugs, smoking, and *Helicobacter pylori* infection. Technically, an endoscopy procedure is performed to confirm the diagnosis of FD, although the utility of endoscopy is minimal in patients with typical symptoms. Because of the incomplete understanding of its mechanisms, FD is hard to treat, even though the condition is usually chronic due to one fluctuating symptom [11,12,13,14,15,16,17].

In addition to numerous conventional treatments for the therapeutic approach of dyspepsia and abdominal pain, many plants have also shown benefits [18,19]. Among them are the species of the genus *Mentha* [20,21].

*Mentha* applications have a millenary tradition and have been used for medicinal effects since ancient civilizations [22]. Due to its therapeutic and medicinal use, the *Mentha* plant is aromatic and perennial, a much-desired herb. *Mentha* components are peppermint essential oil (PO) and non-essential constituents. PO consists of menthol, neomenthol, menthone, and iso-menthone, a blend of volatile metabolites with many different applications, such as antibacterial, antitumor, anti-inflammatory, antiviral, immunomodulatory, antifatigue, neuroprotective, and antioxidant activities. Recent evidence has shown that PO may have pharmacological actions, protecting the liver, skin, kidney, nervous, respiratory, and brain systems; shows gastrointestinal benefits; and can also have hypolipidemic and hypoglycemic effects [23,24].

Due to *Mentha*’s several benefits, this systematic review aims to investigate the effects of this plant on gastrointestinal disorders.

## 2. Results

**Figure 1:**
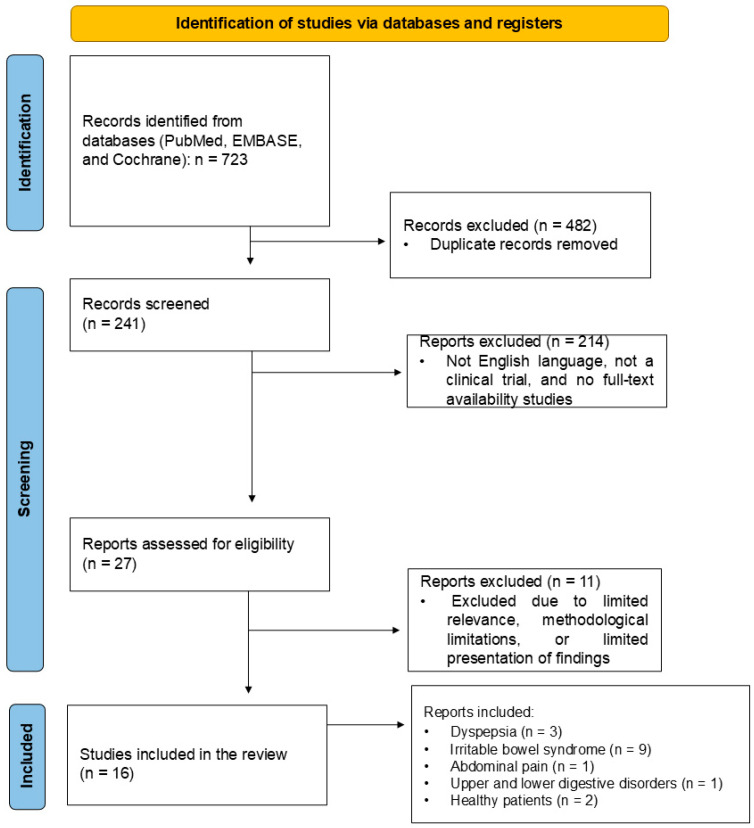
PRISMA flowchart showing the study selection.

This review included sixteen clinical trials, mainly with *Mentha* oil. Studies conducted with a Nutrition Care formula, Carmint, and *Mentha pulegium* were included. Nine studies were performed with IBS patients, three with FD patients, two with healthy patients, one with abdominal pain patients, and one with upper and lower digestive disorders (Figure 1).

The clinical trials that were performed with IBS patients received enteric-coated capsules with around 182 to 225 mg of PO more than once a day for around 4 to 8 weeks. Two of these trials concluded that the PO-treated and placebo groups had equal outcomes for most patients. On the other hand, studies concluded that PO was able to control most IBS symptoms, such as stool frequency and consistency, abdominal pain, and distension. It was also reported that there were changes in the severity of symptoms, reduced abdominal pain or discomfort intensity, and a reduction in the total IBS symptom score (TISS) compared to baseline.

Two studies performed with FD participants received a combination of PO and caraway oil twice daily for about four weeks. The outcomes were significant, as the average pain intensity and sensation of pressure, heaviness, and fullness decreased, indicating a good overall therapeutic effect. One clinical trial with FD patients was performed with *M. pulegium*, and its outcomes were significant, with reductions in stomach pain, upper abdominal dull ache, upper abdominal bloating, and belching.

Among the sixteen clinical trials in the systematic review, two were performed with healthy volunteers who received PO and caraway oil. The PO significantly affected duodenal motility and prolonged orocaecal transit time but did not impact gastric emptying.

One trial investigated the effects of PO in pediatric patients with functional abdominal pain. Its outcome showed that mean pain severity was more significant in the baseline period compared to the time treated with PO, and no adverse events were reported.

One study was performed with patients reporting digestive disorders of the upper and/or lower gastrointestinal tract who received the Nutrition Care Gut Relief Formula powder. The results were significant, as the severity of gastrointestinal symptoms, such as regurgitation, nausea, indigestion, and heartburn, improved.

## 3. Discussion

### 3.1. Mentha Species

Medicinal plant use dates back 60,000 years, even before civilization was built [25]. Although many advancements in modern medicine have been accomplished, traditional medicine holds people’s interest worldwide. Around 80% of the globe’s population utilizes it as their primary healthcare support [26,27].

*M. piperita*, popularly known as peppermint, from the Lamiaceae family, is a herbal medicine that is widely used [28,29]. The genus *Mentha* is distributed throughout North America, Africa, Asia, Australia, and Europe [30,31]. It can be classified into 42 species and 15 hybrids, as well as hundreds of subspecies, cultivars, and varieties. As a natural sterile hybrid of spearmint (*M. spicata*) and watermint (*M. aquatica*), there is also *M. piperita* [22].

Different parts of the plant have been used, as its aerial parts and leaves are the most extensively reported, where the therapeutic value of herbs lies. Moreover, the aerial parts can be dried and transformed into powder or used fresh, extracting an essential oil when subjected to water or steam distillation [22,30]. *M. piperita* oils are extensively used for various purposes as they can be utilized in cosmeceuticals, meals, personal hygiene products, and pharmaceutical items for their aroma and flavoring qualities. It is also used in mouthwashes, aromatherapy, bath preparations, chewing gum, toothpaste, and topical preparations [26,32]. Recently, POs have been highlighted in the path for innovation in therapeutic methods, as alternative medicine represents a contemporary trend with a positive effect on the patient [33].

The plant components are classified as peppermint non-essential and PO [23]. For the non-essential ingredients, it has been observed that *Mentha* has superabundant phenolic compounds, specifically flavonoids, phenols, quinines, terpenes, and polysaccharides [24]. In addition, *Mentha* species contain caffeic acid and its derivatives, cinnamic acid, caftaric acid, oleanolic acid, and ferulic acid. Luteolin and its derivatives acacetin, apigenin, diosmin, thymonin, and salvigenin, which belong to the flavonoids group, have also been identified in these plants, representing around 10-70 components out of the total phenolics, as well as flavonols such as epicatechin, catechin, and coumarins, including scopoletin and esculetin [25].

POs are naturally classified as plants’ volatile secondary metabolites and are characterized by a significant aromatic nature and chemical compositions surrounded by complexity [30]. The species may contain around 300 volatile components, especially esters, alcohols, ketones, oxides, and ethers [25,34]. The monoterpene and sesquiterpenoid fractions of *M. piperita* are at 52% and 9%, respectively. Additionally, the plant also contains aromatic hydrocarbons (9%), aldehydes (9%), miscellaneous (8%), alcohols (6%), and lactones (7%) that appear in smaller proportions. Among the monoterpene components, menthol was the principal constituent at around 35% to 60%. The sequence continues with menthone (2 to 44%), menthyl acetate (0.7 to 23%), 1,8-cineole popularly known as eucalyptol (1 to 13%), menthofuran (0.3 to 14%), isomenthone (2 to 5%), neomenthol (3 to 4%), and limonene (0.1 to 6%). In comparison, β-caryophyllene is the most critical sesquiterpene (1.6 to 1.8%) [30].

Several authors have described the distinct phytochemical composition of different samples of *M. piperita* from various regions. This difference in the PO phytochemical compounds is mainly due to variations in the plant’s harvesting time, stage, drying procedure, and PO extraction methods. Environmental conditions, genetic characteristics, physiological states, locations, and plant evolution also appear to influence the phytochemical variability that exists in the literature [30,35].

**Figure 2:**
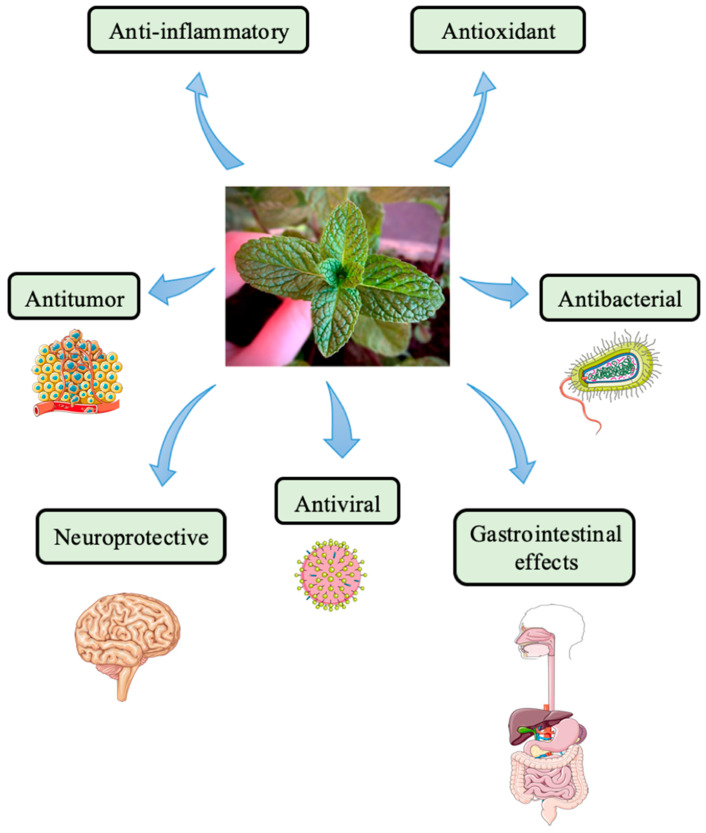
Illustration of *Mentha* species and *Mentha* essential oil effects.

As pointed out, the components of *Mentha* leaves have many health and therapeutic benefits, such as antitumor, antioxidant, antiallergenic, antimicrobial, and immunomodulatory effects, as well as antidepressant effects and gastrointestinal actions [36] (Figure 2). Anti-inflammatory, antiviral, neuroprotective, and antifatigue properties also claim attention. Still, ample scientific evidence indicates that *Mentha* compounds have pharmacological and clinical applications [23,37].

The multiple effects observed using the *Mentha* plant are due to several bioactive compounds (Table 1).

### 3.2. Mentha and Inflammation

The term inflammation defines a broad range of physiological and pathophysiological mechanisms in the human body that primarily aim to prevent the body from diseases and assist in removing dead tissue. At this point, inflammation is a crucial part of the body’s immunological system [84]. Tissue injuries can induce an inflammatory response that leads to the recruitment associated with the proliferation and activation of different variations in immune cells, for example, neutrophils and macrophages, that contribute to tissue repair [85,86]. Acute inflammation is a chain response that includes coordinated cellular and molecular events combined with inflammatory mediators and is self-limited [87,88]. Chronic inflammation occurs if the initiating stimulus persists or the resolution mechanism is disturbed, leading to low-grade inflammation [88,89].

Nuclear factor-kappa B (NF-κB) is a relevant mediator of inflammatory and immune responses [90]. NF-κB is controlled by mitogen-activated protein kinases (MAPKs), and its function is to activate gene transcription in the nucleus, playing an essential role in regulating the inflammatory process since NF-κB stimulates pro-inflammatory cytokines such as interleukin (IL)-6, IL-1β, and tumor necrosis factor-alpha (TNF-α). Activating the MAPK/NF-κB signaling pathway releases cytokines and chemokines essential for immune cell activation. Prostaglandin E_2_ (PGE_2_) and nitric oxide (NO) are synthesized by cyclooxygenase-2 (COX-2) and inducible NO synthase (iNOS), respectively, which are pivotal mediators of the inflammatory response, such as pain, dysfunction, edema, the movement of immune cells, and fever. Therefore, cytokines like ILs and TNF-α are induced by the upregulation of NO, which promotes inflammatory responses and tissue damage [91,92].

Functional gastrointestinal disorders, such as FD, IBS, and inflammatory bowel disease (IBD), can notably affect patients’ quality of life. The symptoms are commonly induced by gastrointestinal infection, diet, alteration of the gut microbiota, stress, psychological factors, and other unknown components, which can lead to a chronic inflammatory process after non-infection or infectious inflammation. Recently, it has been shown that in FD and IBS patients without a history of gastrointestinal infection, the consistent low-grade inflammation of the intestinal mucosa could be related to small-intestinal bacterial overgrowth [93].

IBD is a chronic inflammatory, immune-mediated condition of the gastrointestinal tract. It includes two major types of intestinal disorders: ulcerative colitis (UC) and CD. Most IBD diagnostics are accomplished in early adulthood and can result in a progressive decline in quality of life. Even though the exact cause of IBD has yet to be discovered, its pathophysiology mechanism is complex, and it is hypothesized that a genetically predisposed person who is exposed to yet-to-be-defined environmental conditions, an adverse gut microbiome, and a dysregulated mucosal immune response may develop IBD [94,95,96,97].

Nowadays, the pharmacological management of IBD consists of aminosalicylates, corticosteroids, biological agents, and immunosuppressants. The purpose of the therapy is only to keep the patient’s condition in remission and improve the symptoms related to the disease, rather than focus on modifying or reversing the underlying pathogenic course. Drug therapy in IBD is acknowledged to result in notable adverse effects. Considering this perspective, it is essential to note that new pharmacological approaches aiming for remission and fewer side effects are an advancement in managing IBD and searching for therapeutic options that potentiate the anti-inflammatory effect. On this point, natural products and herbal medicine are consistent options for future therapies, as conventional medical treatment indicates a need for efficacious and safe management [98,99].

A study with ten randomized groups of rats aimed to evaluate the protective effect of *M. longifolia* and eucalyptol, its principal constituent, against acetic acid-induced colitis in rats as a model of human IBD. Measurements and examinations showed positive results for the rat group using 500 mg/kg of sulfasalazine and the rat group using 400 mg/kg eucalyptol, which showed dose-dependent effects. The untreated acetic acid-induced colitis rats presented increased serum IL-6 and TNF-α. In the colonic sections from histopathological examination, it was straightforward to detect severe mucosal ulceration, necrosis, hemorrhage, submucosal edema, inflammatory colonic sections, and goblet cell hyperplasia. Moreover, using *Mentha* also decreased NO production in macrophages and downregulated pro-inflammatory cytokines TNF-α and IL-6, indicating an anti-inflammatory effect [100].

Some authors described that PO, whose main component is menthol, holds anti-inflammatory properties as its oral administration prevents acetic acid-induced colitis in rats and xylene-induced gut inflammation in mice. In vitro, human monocyte inflammatory mediator production was suppressed by menthol. Transient receptor potential cation channels are present in immune cells because it is considered that the anti-inflammatory action of PO may be regulated by the activation of transient receptor potential melastatin 8 (TRPM8), which downregulates chemical-induced colitis in mouse models [101].

Another study investigated the anti-inflammatory effects of PO-loaded alginate microbeads through the examination of an in vivo animal model that consisted of four groups of rats, with six in each, standing for healthy control, disease-induced untreated group, disease-induced drug-treated group (Loperamide 1 mg/kg), and disease-induced PO-treated group. The disease was considered an IBS model induced by 0.5% mustard oil with 30% ethanol. The efficacy of the anti-inflammatory properties was established by observing the animal model stool consistency, histoarchitectural change in the intestine, and body weight loss, demonstrating the effectiveness of PO treatment. The disease-induced PO-treated group showed downregulated expression of IL-1β. It increased the IL-10 concentration in experimental rat plasma, which proves its beneficial anti-inflammatory properties that could also result from the suppressed production of pro-inflammatory mediators like IL-6 and TNF-α [102].

In an investigation with *M. arvensis* essential oil (MAEO) and its effects on lipopolysaccharide (LPS)-induced inflammatory mediators and pro-inflammatory cytokines in RAW 264.7 cells (mouse-derived macrophages), the authors detected that MAEO dose-dependently inhibited LPS-induced PGE_2_ and NO production as a result of further investigation that indicated the suppression of COX-2 and iNOS messenger ribonucleic acid (mRNA) expression by MAEO treatment in RAW 264.7 cells. In addition, it was observed that LPS upregulated the mRNA expression of IL-1β and IL-6. Still, MAEO treatment dose-dependently inhibited IL-6 and IL-1β mRNA expression, reducing the production of IL-1β and IL-6, suggesting MAEO’s anti-inflammatory properties [91].

Some authors performed an experimental 2,4,6-trinitrobenzene sulphonic acid (TNBS)-induced colitis model searching for a reduction in inflammation and colon injury by *M. pulegium* (pennyroyal) phenolic extract, with its main component being rosmarinic acid [103]. This hydroxycinnamic acid is said to be one of the chemical constituents in charge of the anti-inflammatory effect. Mice were randomized and divided into four groups: the control group, the ethanol group (ethanol solution instead of the TNBS solution), the TNBS untreated group, and the TNBS pennyroyal-treated group. Macroscopic and functional signs of colitis injury were evaluated, and the TNBS untreated group showed colon length decrease, diarrhea severity increase, and ulcer increase, combined with a 30% mortality rate; on the other hand, the group treated with pennyroyal extract exhibited a significant reduction in all macroscopic signs of colon injury. Histological features of colitis injury were also checked, with the untreated group presenting severe ulceration and abnormal architecture of crypts and a thinner mucosa with evident immune cell infiltration; on the other hand, colons from the pennyroyal-treated group appeared with slight to moderate lesions combined with minimal crypt modification and less immune cell infiltration. The inflammatory marker analysis showed increased COX-2 and iNOS expression due to colitis induction. Still, with the *M. pulegium* treatment, the expression of both markers was reduced, with iNOS expression appearing to be reduced.

In a study to evaluate the reduction in inflammation and colon injury, colitis was induced in mice by TNBS. The animals were treated with *M. spicata* (spearmint) phenolic extract containing rosmarinic acid. Four experimental groups of mice were randomly distributed, representing the control group, an ethanol group (ethanol solution instead of TNBS solution), a TNBS-untreated group, and a TNBS spearmint-treated group. Macroscopic and histologic examinations were performed, and the TNBS spearmint-treated group presented less severe and shorter colon lesions. The inflammation signs were minimal, with some neutrophilic infiltration. Colon tissue from the TNBS spearmint-treated group showed a high expression of COX-2 but a downregulated expression of iNOS, suggesting that the inflammation may be reduced, in part, by decreasing oxidative stress. One possible mechanism for spearmint extract modulation is the expression of iNOS, regarding its action attenuating the inflammatory process and following rosmarinic acid’s beneficial effects [98].

From this perspective, *M. piperita* essential oil and extracts also exhibit anti-inflammatory actions that may decrease inflammation and prevent chronic diseases. *M. piperita* ethanolic extract reduced the LPS-induced NO production and suppressed pro-inflammatory cytokines in the murine macrophage cell line RAW 264.7 treated with LPS [31].

Inflammatory processes are closely linked with the production of free radicals, such as reactive oxygen species (ROS). These processes aggravate each other’s harmful effects on the organism [104].

**Figure 3:**
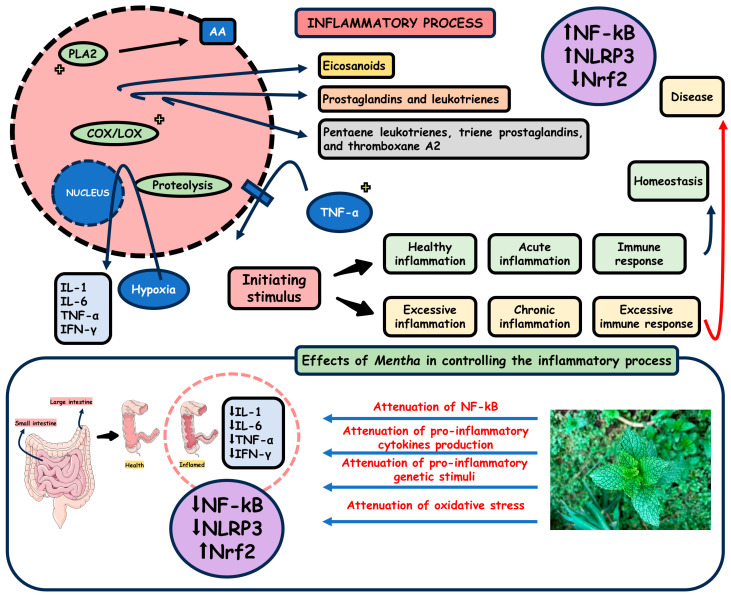
*Mentha* has anti-inflammatory effects. Abbreviations: PLA2, phospholipase A2; AA, arachidonic acid; COX, cyclooxygenase; LOX, lipoxygenase; IL, interleukin; TNF-α, tumor necrosis factor alpha; IFN-γ, interferon gamma; NF-κB, nuclear factor-kappa B; NLRP3, NOD-like receptor family pyrin domain containing 3; Nrf2, nuclear factor erythroid 2-related factor 2.

Figure 3 demonstrates the numerous anti-inflammatory effects of *Mentha*.

### 3.3. Mentha Species and Oxidative Stress

The term antioxidant refers to substances or molecules that can delay or even prevent the irreversible damage of other macromolecules due to metabolite instability in the human body and offer health benefits. The supply of antioxidants is critical to reacting with and neutralizing ROS, including free radicals produced by several metabolic reactions during physiological mechanisms [105,106]. Oxidative stress is caused by an imbalance between the production of reactive species and the antioxidant micro-ecosystem that supports the excessive generation of pro-oxidants, which could lead to alterations of biological systems. It is a cause of an extensive range of diseases, for example, chronic obstructive pulmonary disease, neurodegenerative diseases, cardiovascular diseases, cancer, chronic kidney disease, and IBD [107,108].

The physiological levels of ROS are essential to maintaining the signaling pathways responsible for controlling cellular processes, including inflammation, proliferation, differentiation, and apoptosis. However, dysregulated ROS release can promote overstimulation of these pathways, leading to ROS-associated disease [109,110].

The gastrointestinal tract is considered one of the primary sources of ROS and, on the other hand, a primary target. Even though the epithelium functions as a physical and antimicrobial barrier, material ingestion and enteric pathogens can contribute to the initiation of inflammation by stimulating the generation of pro-inflammatory cytokines; nevertheless, free radical signaling is non-specific, which can lead to unwanted tissue damage and, consequently, start inflammatory processes [111].

Acute and chronic disorders in the gastrointestinal tract in animal models and humans are marked by increased ROS production or the downregulation of endogenous antioxidant components [112]. Different studies have documented that experimental models of colitis and IBD patients have prominent oxidative stress processes and a reduction in the scavenging of free radicals. Additionally, severe oxidative stress markers are evident, and inflammatory response enzymes that also participate in the production of ROS are upregulated [111].

Antioxidants can be endogenous or exogenous. Endogenous antioxidants include catalase, superoxide dismutase, and glutathione peroxidase. Exogenic antioxidants include vitamins, flavonoids, and minerals [110]. The Lamiaceae family is one of the most significant medicinal plant families [106].

Many studies have evaluated *M. spicata*’s antioxidant activity by measuring its effectiveness in scavenging free radicals. The essential oil from the leaves of this plant exhibited potent radical scavenging actions, as menthone and pulegone are the main volatiles of *M. spicata* essential oil [49].

Recent studies have shown *M. piperita*’s antioxidant activities. The antioxidant DPPH (2,2-diphenyl-1-picrylhydrazyl) free radical scavenging of essential oil and different extracts was observed. The inhibition percentages have shown variations, but the highest values were obtained for crucial oil (92.8 ± 6.8%) and chloroform extract (91.8 ± 5.8%), and the lowest value was for aqueous extract (70.3 ± 6.1%). The main compounds in PO are menthol, L-menthol, eucalyptol, and neo-menthol [113].

The DPPH assay was used to estimate the antiradical activity of *M. pulegium* essential oil, with Vitamin C as the standard reference. Concentrations of *M. pulegium* essential oil at around 40 to 60 mg/mL have shown the inhibition of approximately 80% of free radicals in DPPH free radical scavenging tests. Pulegone is a ketone monoterpene, and this oil’s primary chemical compound may be an antioxidant component [114].

Recent investigations have shown that gut microbiota dysbiosis is directly linked to bowel inflammation and oxidative-initiating events. Improving the gut microbiota is crucial for understanding and treating bowel inflammatory conditions [115,116]. Due to the *Mentha* species’ anti-inflammatory and antioxidant effects, it is possible to postulate that these plants can be of great importance in the therapeutic approach to gastrointestinal diseases [117,118,119].

**Figure 4:**
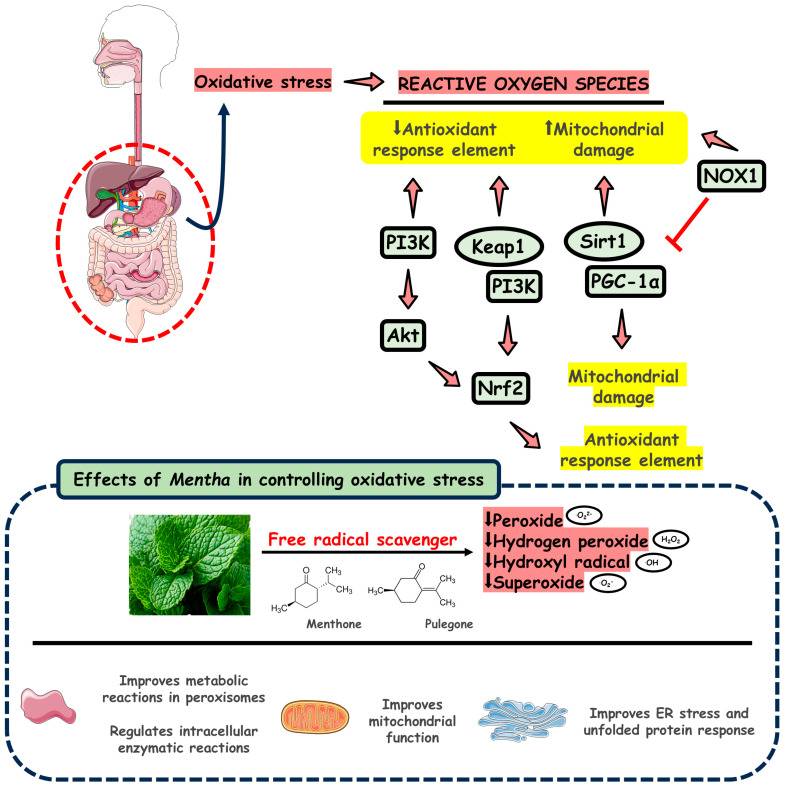
Antioxidant effects of *Mentha*. Abbreviations: NOX1, NADPH oxidase 1; PI3K, phosphatidylinositol 3-kinase; Akt, protein kinase b; Nrf2, nuclear factor erythroid 2-related factor 2; Sirt1, Sirtuin 1; PGC-1α, peroxisome proliferator-activated receptor gamma coactivator 1-alpha; Keap1, Kelch-1 ECH-associated protein 1; ER, endoplasmic reticulum.

Figure 4 shows the multiple antioxidant effects of *Mentha*.

### 3.4. Mentha, Abdominal Pain, and Dyspepsia: The Results of Clinical Trials

In addition to producing effects in animals, *Mentha* species also show positive effects in humans, as shown in Table 2. The bias risk for these studies is shown in Table 3.

One interesting study showed the tolerability and efficacy of an enteric-coated peppermint oil formulation (Colpermin) in treating IBS. The symptoms were evaluated before and after 1 month of intervention. It showed that the unique formulation with delayed release ensured the active component reached the colon in an unmetabolized state and prevented esophageal reflux and heartburn. Although the results confirm the efficacy of peppermint oil, the short duration of the study and the broad age range may be potential biases in the interpretation of the results [120].

Another study also investigated the clinical usefulness and efficacy of pH-dependent, enteric-coated peppermint oil capsules (Colpermin) in treating IBS symptoms in children. The results are interesting, as peppermint oil reduced the severity of pain associated with IBS. However, the sample size was small, the duration of the study was short, and most participants were white, which are possible biases in the study [121].

In another trial, the authors studied the tolerability and efficacy of a fixed combination of caraway and peppermint oil in patients with FD. The treatment group’s intensity of pain, sensation of pressure, heaviness, and fullness were statistically and clinically relevant compared to the placebo. The short duration of the study, the significant losses of 17% of participants at the end of the study, and the combined formulation, as there is no certainty of which component led to the positive results, could be biases in interpreting the study [122].

Another study aimed to investigate the effect of caraway oil and peppermint oil on gastrointestinal motility in healthy participants. PO and caraway oil could be used as a gastrointestinal muscle relaxant, especially by menthol’s spasmolytic action, and as a fundus relaxant that may prevent the fullness feeling, which patients with FD experience. Even though the results are significant, the sample size was small; the intervention was performed in healthy volunteers, the study’s short duration, and the lack of information about the participants’ race introduce some biases into the interpretation of the results. Additionally, the fact that it did not have an appropriate blinding of participants and investigators may compromise the fidelity of the result interpretation and, even more, the combined formulation cannot correctly distinguish which component caused specific effects [123].

The effects of caraway oil and peppermint oil on gastroduodenal motility were also evaluated in 24 healthy volunteers. The frequency and amplitude of contractions were measured before and after administering the substance under study. The results showed that the intraduodenal application of the combined formula could reduce motility in the gastric corpus and antrum. However, the small number of volunteers, the combined formulation, and especially the short duration of the study performed in a day could bias the interpretation of the results [124].

One critical study demonstrated the efficacy of Carmint on abdominal pain and bloating in patients with IBS. Carmint is a herbal medicine combining *Melissa officinalis*, *Mentha spicata*, and *Coriandrum sativum* extracts. The results showed that the frequency and severity of pain and bloating were lower at the end of the intervention. The allocation blinding and duration of the study were performed correctly. Notwithstanding that, the sample size was small, and using a combined herbal medicine in addition to psyllium and loperamide may be biased when interpreting the results [125].

Another study aimed to investigate the effectiveness of pH-dependent, enteric-coated PO capsules in patients with symptoms of IBS. The results are interesting, showing that the group treated with peppermint oil had a statistically significant improvement in IBS symptoms, and it also showed that the beneficial effect of peppermint oil lasted for 1 month after the therapy ended in more than half of the treated participants. It is due to peppermint’s relaxing effect on the intestinal smooth muscle because of menthol’s actions in calcium movement across the cell membrane, also explained by Goerg et al., which also discussed peppermint oil’s spasmolytic actions. However, the duration of the study was short, with a wide age range, and the lack of race information could cause biases in interpreting the results [126].

One crucial trial also evaluated the effect of enteric-coated, delayed-release peppermint oil capsules in outpatients with IBS to enhance quality of life and relieve symptoms. The follow-up was performed four times during the study, at the beginning and weeks 1, 4, and 8. It was concluded that peppermint oil effectively improved abdominal pain and discomfort. However, the age range was broad and included race information, and the losses of participants for follow-up were significant, almost 30%, which is a possible bias in this study [127].

Another clinical trial investigated the effectiveness of a novel formulation of triple-coated microspheres of solid-state with highly purified peppermint oil designed to be released in the small intestine in patients with non-constipated IBS. The treated group showed a statistically significant reduction in mean TISS and decreased abdominal pain and discomfort compared to baseline. Even considering the promising results, the sample size was relatively small, the study duration was limited to 28 days, and the number of female participants was higher and predominantly white, which may compromise the interpretation of the results [128].

The efficacy of a fixed combination of peppermint oil and caraway oil (Menthacarin) was evaluated in patients with FD symptoms consistent with postprandial distress syndrome and epigastric pain syndrome. At the end of the study, the results showed that Menthacarin effectively alleviates pain and discomfort and improves the quality of life. Even though the study duration was short, the losses of participants were significant, almost 24%, with biased results [129].

In another study, the authors investigated the efficacy of *M. pulegium* in treating FD. The results showed its effectiveness because stomach pain, dull abdominal ache, and upper abdominal bloating decreased in the treated group compared to the baseline. However, during the intervention, participants took a 40 mg famotidine tablet once a day, representing a bias for the study [130].

In this study, the authors conducted a single-arm trial to test the tolerability and efficacy of the herbal Nutrition Care Gut Relief Formula that contains curcumin, *Aloe vera*, guar gum, slippery elm, glutamine, and peppermint oil in participants with upper and/or lower gastrointestinal tract disorders. It showed improvement in heartburn, indigestion, constipation, and abdominal pain in most of the participants. Despite the promising results, several biases must be considered, such as the lack of a control group during all the intervention periods, no allocation blinding, and many female volunteers. Furthermore, the formula with different components cannot secure which constituent was responsible for each effect [131].

In contrast with most trials, this study investigated the safety and efficacy of small-intestinal release peppermint oil in participants with IBS. It evaluated the effects of targeted ileocolonic-release peppermint oil. However, the results concluded that neither ileocolonic-release nor small-intestinal release could produce statistically significant reductions in abdominal pain response, considering that a responder is a participant with at least a 30% decrease in the weekly average of worst daily abdominal pain compared with baseline for at least 50% of the intervention period. However, the minor intestinal release did improve abdominal pain and discomfort. Moreover, some biases must be taken into consideration, such as the sample population being relatively young and the female gender and white race being predominant [21].

This other trial also analyzed peppermint oil efficacy compared to a placebo in treating IBS symptoms. The results showed no significant difference between the treated group and placebo, as both improved the IBS-SSS score (IBS severity scoring system). However, most participants clinically obtained meaningful symptom improvement with either therapy. The trial also concluded that the PO-treated group had more side effects. The sample population was predominantly female and white, and the placebo group was double the size of the treated group, which could lead to biases in interpreting the results [132].

Gastrointestinal disorders, such as IBS, frequently impose a socioeconomic burden, and this trial evaluated the cost-effectiveness of small-intestinal release peppermint oil in patients with IBS. The study concluded that small-intestinal PO seems cost-effective from a societal and healthcare perspective. The participants were white, relatively young, and predominantly female; the age range was wide, and there were significant losses at the end of the study, which may be biased when interpreting the results [133].

In this critical trial, the participants took enteric-coated PO, and the aim was to evaluate the effect of menthol on gut motility and transit in children with functional abdominal pain. The results showed that the mean pain severity improved during the treatment compared to baseline, and it was also concluded that the whole gut transit time was unaffected. Still, the entire gut mean peak contraction amplitude seemed to reduce. However, the duration of the study was minimal; the sample size was small and predominantly female, which could have biased the results [134].

## 4. Materials and Methods

### 4.1. Focal Question

This systematic review was performed to answer the question, “Can *Mentha* species or *Mentha* essential oil produce beneficial effects on gastrointestinal disorders in humans?”

### 4.2. Language

Only studies published in English were selected.

### 4.3. Databases

This review includes studies in the MEDLINE–PubMed (National Library of Medicine, National Institutes of Health), COCHRANE, and EMBASE databases. The mesh terms used were as follows: *Mentha* or *Mentha* essential oil, dyspepsia, abdominal pain, diarrhea, abdominal motility, irritable bowel syndrome, or gastrointestinal disorders. These descriptors helped identify studies related to *Mentha* and its gastrointestinal effects. The Preferred Reporting Items for a Systematic Review and Meta-Analysis (PRISMA) guidelines guided the search for the included studies [135]. Two experienced authors searched for the included studies (L.F.L. and S.M.B.). Discrepancies between them were resolved by a third author (V.E.V.).

### 4.4. Study Selection

Abstracts, conferences, letters to editors, and other sources were consulted but not included. Moreover, other relevant studies about *Mentha* species and human health were evaluated to help in the Introduction and Discussion Sections. Only human interventional studies were included in this systematic review. Editorials, reviews, studies not in English, case reports, and poster presentations were excluded. Figure 1 shows the flowchart of the study selection according to PRISMA guidelines.

### 4.5. Data Extraction

We did not restrict the search period. The data were extracted using the PICO (Population, Intervention, Comparison, and Outcomes) format. The retrieved articles are shown in Table 1.

### 4.6. Quality Assessment

To evaluate the risk of bias related to the selection of the studies, we consulted the Cochrane Handbook for systematic reviews of interventions [136]. Two reviewers (L.F.L. and S.M.B.) independently performed the risk of bias analysis. In discrepancies, a third researcher (V.E.V.) was consulted to resolve inconsistencies. Additionally, we identified factors that could affect the overall body of evidence, such as publication bias and selective reporting within studies. The risk of bias assessment evaluators underwent appropriate training sessions to ensure consistency and accuracy in their evaluations.

## 5. Conclusions and Future Research Perspectives

In conclusion, *Mentha* and its PO can promote notable effects in reducing pro-inflammatory and pro-oxidative actions, better controlling abdominal pain and discomfort in IBD patients, and improving symptoms in FD and functional abdominal pain patients. *Mentha* and bioactive compounds such as menthol, neomenthol, menthone, iso-menthone, flavonoids, phenols, quinines, terpene, caffeic acid (and its derivatives), cinnamic acid, caftaric acid, oleanolic acid, ferulic acid, luteolin and its derivatives, epicatechin, catechin, and coumarins, including scopoletin and esculetin, can be used in many other applications, such as antibacterial, antitumor, anti-inflammatory, antiviral, immunomodulatory, antifatigue, neuroprotective, and antioxidant activities. Even though it must be taken into consideration that *Mentha*’s components can ameliorate the symptoms of these diseases and even reach the standard of being considered for use in clinical treatments, more clinical studies must be performed to evaluate the safety of doses and formulations for good availability to achieve even better outcomes.

Due to the increasing search for natural compounds in the therapeutic approach to numerous diseases, including gastrointestinal disorders, there have been several studies on plants and their bioactive compounds. *Mentha* has been used for thousands of years to improve digestive system disorders. Shortly, many technological advances are expected in the use of *Mentha*. New technologies, such as nanotechnology, can be developed to increase the bioavailability of its compounds, improving therapeutics and facilitating the acquisition of these compounds. In addition, new formulations can use other compounds with synergistic effects to improve gastrointestinal effects.

## Figures and Tables

**Table 1:** Bioactive compounds found in *Mentha* species.

Bioactive Compounds	Molecular Structures	Plant Parts	Health Effects	References
Acacetin	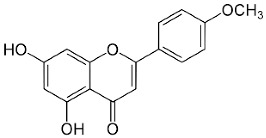	Flowering aerial parts and leaves	Anti-inflammatory, anticancer, anti-obesity, anti-diabetic, antioxidant, neuroprotective, and cardioprotective	[38,39,40,41,42]
β-caryophyllene	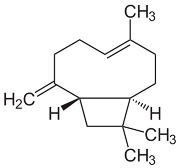	Flowering aerial parts and leaves	Antioxidant, immunomodulatory, anti-inflammatory, anti-diabetic, antitumor, and gastroprotective	[43,44]
Caffeic acid	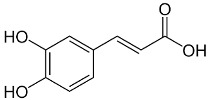	Flowering aerial parts and leaves	Antioxidant, anticancer, antibacterial, antiviral, anti-inflammatory, anti-diabetic, and anti-dyslipidemia	[45,46,47,48,49]
Carvone	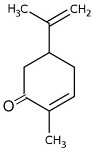	Flowering aerial parts and leaves	Antibacterial, antifungal, anti-parasitic, antioxidant, anti-inflammatory, anticancer, and anti-diabetic	[49,50,51]
Epicatechin	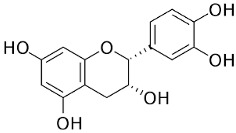	Flowering aerial parts and leaves	Antihypertensive, vasorelaxant, antioxidant, anti-inflammatory, anti-diabetic, anti-dyslipidemia, and anti-atherosclerotic, hepatoprotective, and neuroprotective	[49,52,53,54]
Isomenthol	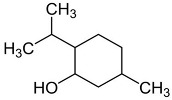	Flowering aerial parts and leaves	Thermogenic, anti-spasmodic, antioxidant, hepatoprotective, anti-inflammatory, and antibacterial	[43,55]
Limonene	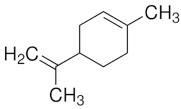	Flowering aerial parts and leaves	Antioxidant, anti-diabetic, anticancer, anti-inflammatory, cardioprotective, gastroprotective, hepatoprotective, immunomodulatory, and anti-fibrotic	[43,56,57,58]
Luteolin	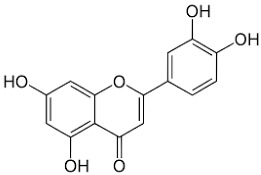	Flowering aerial parts and leaves	Antioxidant, anti-inflammatory, anticancer, and antiviral	[49,59,60,61,62]
Menthofuran	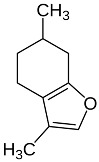	Flowering aerial parts and leaves	Antidiarrheal and gastroprotective	[43,63,64]
Menthol	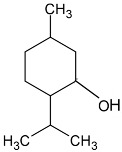	Flowering aerial parts and leaves	Analgesic, antibacterial, antifungal, anesthetic, immunomodulatory effects, anti-inflammatory, and anticancer	[43,65,66,67,68]
Menthone	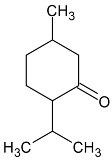	Flowering aerial parts and leaves	Antiviral, anti-inflammatory, antioxidant, antidepressant, antifungal, anticancer, and antibacterial	[43,69,70]
Menthyl acetate	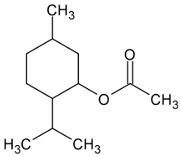	Flowering aerial parts and leaves	Anti-inflammatory, analgesic, antifungal, antiviral, and antimicrobial	[71,72,73]
Neomenthol	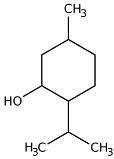	Flowering aerial parts and leaves	Cooling–soothing effects, treatment of sore throat and mouth irritations	[43,74]
Pulegone	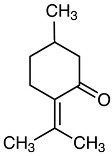	Flowering aerial parts and leaves	Antioxidant, antimicrobial, antifungal, antiviral, antibacterial, antihistaminic, and anti-inflammatory	[43,75,76]
Rosmarinic acid	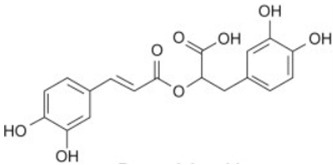	Flowering aerial parts and leaves	Antioxidant, antibacterial, antiviral, anti-inflammatory, anti-hyperglycemic, analgesic, hepatoprotective, immunomodulatory, anticancer, cardioprotective, and neuroprotective	[49,77,78,79,80]
1,8-cineole (eucalyptol)	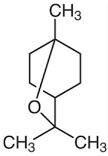	Flowering aerial parts and leaves	Antimicrobial, anti-inflammatory, antioxidant, and anti-fibrotic	[43,81,82,83]

**Table 2:** Studies showing the effects of *Mentha* or *Mentha* oil.

Ref	Model/Country	Population	Intervention/ Comparison	Outcomes	Side Effects
[120]	Randomized, prospective, double-blind, placebo-controlled clinical study, single-center/Taiwan.	110 patients, 66 ♂, 44 ♀, 18–70 y, with active symptoms of IBS.	Participants received enteric-coated capsules containing 187 mg of PO (n = 52) or a placebo (n = 49), 3 to 4 times daily, 15–30 min before meals/4 weeks.	PO controlled most symptoms of IBS (abdominal pain, distension, stool frequency and consistency, borborygmi, and flatulence) in about 78% of patients (*p* < 0.05).	Heartburn was present at the beginning of treatment, and there was a mild skin rash over both forearms (PO group).
[121]	Randomized, double-blind, controlled trial, multicenter/USA.	50 patients, 40% ♂, 60% ♀, 8–17 y, previously diagnosed with IBS.	Participants received enteric-coated capsules containing 187 mg of PO or a placebo. >45 kg = 2 capsules 3 times daily or 30–45 kg = 1 capsule 3 times daily/2 weeks.	PO group: 76% of the patients reported changes in the severity of symptom scale (*p* < 0.001), and the severity of pain symptoms was significantly lower (*p* < 0.03). There are no changes in abdominal rumbling, distention, belching, gas, or heartburn.	No side effects were reported.
[122]	Randomized, double-blind, parallel-group, multicenter/Germany.	96 patients, 32 ♂, 64 ♀, at least 18 y with a secured diagnosis of FD.	Participants received enteric-coated capsules containing 90 mg PO and 50 mg caraway oil (n = 48) or a placebo (n = 48), two capsules daily (in the morning and at lunchtime) for 28 days.	The average pain intensity decreased by 40% (*p* = 0.0003), and the sensations of pressure, heaviness, and fullness decreased by 43.5% (*p* = 0.0005) in the treated group.	Neck and shoulder pain, hemorrhoids, bronchitis, influenza-like symptoms, mild eructation, nausea, and vomiting.
[123]	Clinical trial, single-center/Germany.	12 patients, 6 ♂, 6 ♀, 24–51 y, healthy volunteers.	On day 1, a placebo was given; for the next 4 days, the substances 90 mg PO, 50 mg caraway oil, 10 mg cisapride, and 10 mg n-butyl scopolamine were studied in a randomized sequence, with a washout phase of 2 days after each investigation.	Orocaecal transit time was prolonged by PO (*p* = 0.004), and caraway oil was not significant. No effects on gastric emptying, inhibition of gallbladder emptying.	Eructation with a peppermint taste. One case was associated with mild heartburn that rapidly disappeared.
[124]	Randomized, prospective, controlled, double-blind, two-period crossover trial, single-center/Germany.	24 patients, 12 ♂, 12 ♀, 21–42 y, healthy volunteers.	Participants received the lipophilic carrier/control + 90 mg PO or a placebo, control + 50 mg caraway oil, or control + 50 mg hydrophobic phase in 1 day.	Intraduodenal application of 90 mg PO and 50 mg caraway oil affects duodenal motility and reduces motility in the gastric antrum and corpus.	No adverse events were observed during the study.
[125]	Randomized, double-blind, placebo-controlled, multicenter/Iran.	32 patients, 18 ♂, 14 ♀, 18–65 y with IBS diagnosed using the Rome II criteria.	Participants received 30 drops of Carmint (combination of *Melissa officinalis*, *Mentha spicata*, and *Coriandrum sativum*) (n = 14) or a placebo (n = 18) 3 times a day after each meal. They also received loperamide (2 mg/2x/d) (n = 10) or one spoonful of psyllium powder 1x/d (n = 22) for 8 weeks.	Carmint group: the average abdominal pain/discomfort severity and frequency were significantly lower (*p* = 0.016), and the average bloating severity score (*p* = 0.02) at the end of the treatment.	Anal pain before defecation, malaise, anxiety/chest pain/sweating.
[126]	Randomized, prospective, double-blind, placebo-controlled clinical trial, single-center/Italy.	According to the Rome II criteria, 50 patients, 12 ♂, 38 ♀, 20–60 y with IBS.	Participants received capsules with 225 mg PO + 45 mg Natrasorb (n = 24) or a placebo (n = 26). Two enteric-coated capsules twice a day, before meals, for 4 weeks.	75% of patients in the PO group showed a > 50% reduction in basal TISS (*p* < 0.009), and a beneficial effect persisted after the other 4 weeks (*p* < 0.05).	Intense heartburn and a minty taste in his mouth.
[127]	Randomized, double-blind, placebo-controlled clinical trial, single-center/Iran.	60 patients, 15 ♂, 45 ♀, 22–48 y with diagnosed IBS by Rome II criteria	Participants took one enteric-coated capsule of 187 mg PO or a placebo, 3 times daily, 30 min before each meal for 8 weeks.	42.5% of PO group patients were free from abdominal pain or discomfort (*p* < 0.001), and the intensity of the pain was also significantly reduced (*p* < 0.001).	Heartburn, headache, and dizziness.
[128]	Randomized, double-blind, placebo-controlled clinical trial, multicenter/USA.	72 patients, 18 ♂, 54 ♀, 18–60 y, who met Rome III criteria for IBS-M or IBS-D.	Patients were instructed to take two capsules of 180 mg PO or a placebo 3 times daily between 30 and 90 min before meals for 4 weeks.	The PO group showed a 40% reduction in the TISS from baseline (*p* = 0.0246) and a 21% decrease in abdominal pain and discomfort (*p* = 0.0138).	Flatulence, dyspepsia, and gastroesophageal reflux.
[129]	Randomized, prospective, double-blind, parallel-group, placebo-controlled clinical trial, multicenter/Australia and Germany.	114 patients, 41 ♂, 73 ♀, 32–62 y with FD symptoms consistent with EPS and PDS.	Participants received 90 mg PO + 50 mg caraway oil or a placebo, two capsules per day to be taken with liquids in the morning and at noon, before meals for 4 weeks.	88% of the patients from the treated group showed any improvement (*p* = 0.0001), and an overall therapeutic effect for 51.7% of patients was assessed to be very good (*p* < 0.0001).	Eleven patients from the treated group experienced adverse events, which were not reported in the study.
[130]	Randomized, 2-arm, double-blind, placebo-controlled parallel-group, single-center/Iran.	One hundred patients, 48 ♂, 52 ♀, aged 25–56 y, fulfilled Rome III criteria for FD.	Participants received 330 mg of *Mentha pulegium* extract powder or a placebo three times a day and one famotidine 40 mg tablet daily for 8 weeks.	*Mentha pulegium* significantly reduced the total dyspepsia score (Hong Kong dyspepsia index) (*p* = 0.011) and decreased stomach pain, upper abdominal bloating, dull ache, and belching.	No adverse events were reported.
[131]	Single-arm, pre-post study, single-center/Australia.	Forty-three patients, 24% ♂, 76% ♀, mean age of 50 y, experiencing one or multiple symptoms at least once a week for 3 months, such as heartburn, reflux, nausea, regurgitation, abdominal pain, bloating, diarrhea, constipation, and IBS.	After a 4-week control phase (0 g/d), patients received 5 g/d of the Nutrition Care Gut Relief Formula powder (a combination of curcumin, *Aloe vera*, guar gum, slippery elm, glutamine, and PO) for 4 weeks, followed by 10 g/d for another 4 weeks and more 4 weeks of the patient’s preferred dose (0/5/10 g/d). The powder is to be taken mixed with water or with food.	The formula improved the GI symptoms’ severity (56% to 62%) and frequency (64%) as indigestion, heartburn, regurgitation, and nausea (*p* < 0.001) and also the frequency and severity abdominal pain (62%) and troublesome flatulence (58%) (*p* < 0.0001).	No bothersome adverse events were reported.
[21]	Randomized, double-blind, placebo-controlled trial, multicenter/The Netherlands.	189 patients, 42 ♂, 147 ♀, 18–70 y, fulfilling the Rome IV criteria for IBS.	Participants received 182 mg of small-intestinal release PO or 182 mg of ileocolonic-release PO or a placebo, three capsules daily, 30 min before breakfast, lunch, and dinner, for 8 weeks.	The proportion of patients who reported abdominal pain did not differ drastically between groups: 46.8% in the small-intestinal release group (*p* = 0.170) and 41.3% in the ileocolonic-release peppermint oil group (*p* = 0.385), compared with 34.4% in the placebo.	Heartburn, belching, headache, nausea, abdominal cramps, and altered anal sensation or sensitive urethra.
[132]	Randomized, double-blind, placebo-controlled trial, single-center/USA.	133 patients, 35 ♂, 98 ♀, mean age in the early 40s, with IBS diagnosed by the Rome IV criteria.	Patients received 180 mg enteric-coated PO capsules or a placebo group 3 times a day for 6 weeks.	A substantial mean improvement in the IBS-SSS score for both groups showed no significant difference in scores between the two groups (*p* = 0.97). Both groups had equal benefits for most patients.	Belching and heartburn.
[133]	Randomized, double-blind, placebo-controlled trial, multicenter/The Netherlands.	126 patients, 26 ♂, 100 ♀, 18–75 y, fulfilling the Rome IV criteria for IBS.	Patients received a placebo or 182 mg small-intestinal release PO for 8 weeks.	The small-intestinal release PO is more effective at a lower cost in 46% of the simulations, but at a higher cost in 31%, while it is inferior in 18% (less effective and higher costs).	No adverse events were reported.
[134]	Randomized trial, single center/USA.	30 patients, 9 ♂, 21 ♀, 7–12 y, with functional abdominal pain (pediatric Rome III criteria).	At visit 1, patients received a WMC. After 1 week, they received 180 mg, 360 mg, or 540 mg of enteric-coated PO for 1 week, during which time the WMC test was repeated.	Small-intestinal transit time was slower (*p* = 0.066). Stomach (*p* = 0.02) and duodenal (*p* = 0.039) contraction frequency increased with increasing peppermint oil dose, as well as ileum (*p* = 0.028) and colonic (*p* = 0.075) mean contraction amplitude.	No adverse events were reported.

Abbreviations: GI: gastrointestinal; IBS: irritable bowel syndrome; FD: functional dyspepsia; EPS: epigastric pain syndrome; PDS: postprandial distress syndrome; IBS-M: mixed IBS; IBS-D: diarrhea-predominant IBS; TISS: total IBS symptom score; WMC: wireless motility capsule; IBS-SSS: IBS severity scoring system; PO: peppermint essential oil.

**Table 3:** A descriptive table of the biases of the included randomized clinical trials.

Study	Question Focus	Appropriate Randomization	Allocation Blinding	Double- Blind	Losses (>20%)	Prognostic or Demographic Characteristics	Outcomes	Intention to Treat Analysis	Sample Calculation	Adequate Follow-Up
[120]	Yes	Yes	Yes	Yes	No	No	Yes	No	No	Yes
[121]	Yes	Yes	Yes	Yes	No	No	Yes	No	Yes	Yes
[122]	Yes	Yes	Yes	Yes	No	Yes	Yes	Yes	No	Yes
[123]	Yes	No	No	No	No	No	Yes	No	No	Yes
[124]	Yes	Yes	Yes	Yes	No	No	Yes	Yes	No	Yes
[125]	Yes	Yes	Yes	Yes	No	Yes	Yes	Yes	No	Yes
[126]	Yes	Yes	Yes	Yes	No	No	Yes	Yes	Yes	Yes
[127]	Yes	Yes	Yes	Yes	Yes	Yes	Yes	No	No	Yes
[128]	Yes	Yes	Yes	Yes	No	Yes	Yes	Yes	No	Yes
[129]	Yes	Yes	Yes	Yes	Yes	Yes	Yes	Yes	No	Yes
[130]	Yes	Yes	Yes	Yes	No	Yes	Yes	Yes	No	Yes
[131]	Yes	No	No	No	No	No	Yes	No	Yes	Yes
[21]	Yes	Yes	Yes	Yes	No	Yes	Yes	Yes	No	Yes
[132]	Yes	Yes	Yes	Yes	No	No	Yes	Yes	No	Yes
[133]	Yes	Yes	Yes	Yes	Yes	Yes	Yes	Yes	No	Yes
[134]	Yes	Yes	No	No	No	Yes	Yes	No	Yes	Yes

## Data Availability

This study did not create or analyze new data, and data sharing does not apply to this article.
